# Supplementary material for: Barriers and facilitators to physicians’ telemedicine uptake during the beginning of the COVID-19 pandemic
Source: PLOS Digit Health. 2025 Apr 8;4(4):e0000818. doi: 10.1371/journal.pdig.0000818 (PMC11977993; doi:10.1371/journal.pdig.0000818)
Supplement: S1 Table — (DOCX) [file pdig.0000818.s001.docx]

**S1 Final List of Barriers and Facilitators**

| Barriers | |
| --- | --- |
| Code | Description |
| Lack of Patient Access to Technology | Concern for inability to access technology necessary for telemedicine (e.g., lack of high-speed internet in rural locations or inability to purchase a smartphone/laptop |
| Insufficient Insurance Reimbursement | Concern for inadequate reimbursement for telemedicine services from insurance, Medicare, etc. |
| Higher Cost for Patients | Concern for financial costs associated with obtaining, implementing, and maintaining telemedicine equipment for patients |
| Higher Cost for Providers | Concern for financial costs associated with obtaining, implementing, and maintaining telemedicine equipment for providers |
| Insufficient Telemedicine Training | Statements endorsing a lack of training for telemedicine services or policies and procedures |
| Diminished Doctor-Patient Relationship | Concern for how lack of in person interaction might affect the doctor-patient relationship |
| Inefficient Use of Time | Statements regarding wasted time (e.g., setting up or troubleshooting telemedicine issues) |
| Lack of Leadership Support | Statements regarding a general dislike/distrust of telemedicine by one's employer |
| Diminished Quality of Delivered Care | Includes concerns regarding efficacy, effectiveness, and the need for more research on telemedicine |
| Ethical Issues | Statements questioning the ethicality of telemedicine |
| HIPAA Requirements | Statements regarding HIPAA regulations governing its use |
| Legal Issues | Statements regarding the legality of telemedicine |
| Unsupportive Prescription Regulations | Statements endorsing difficulty navigating prescriptive regulations or stating the prescription regulations are too burdensome |
| Higher Risk to Patient Safety | Expressions of worry for remote clients who may be in crisis or at risk of harm to self or others; also includes concerns related to patient privacy as a safety risk |
| Inadequate Video/Audio Quality of Telemedicine Technology | Includes statements that the technological requirements for telemedicine are currently inadequate or unreliable (e.g., poor connectivity or quality) as well as statements regarding the need for troubleshooting that do not reference "time". |
| Potential for Medical Errors | Statements surrounding the increased likelihood for medical errors as a result of remote medical treatment |
| Inadequate Patient Technological Literacy* | Statements regarding patient's lack of knowledge for how to operate technology or telemedicine applications |
| Not Suitable for Certain Patients or Types of Care* | Statements regarding how telemedicine is either not used or especially difficult to use for certain patients or types of care |
| Facilitators | |
| Better Access to Care | Statements regarding how telemedicine allows providers to assist patients who would otherwise not be able to be seen |
| Lower Cost for Patients | Statements regarding reduced cost for the patient |
| Lower Cost for Providers | Statements regarding reduced cost for the provider or increased revenue for the provider |
| Telemedicine Training I have Received | Statements in which the provider endorsed comfortability with telemedicine as a specific result of having been trained in telemedicine services |
| Better Doctor-Patient Relationship | Statements regarding how telemedicine positively effects the doctor-patient relationship |
| Efficient Use of Time | Statements regarding how telemedicine saves either the patient or provider time |
| Effectiveness | Statements indicating more effective care as a result of telemedicine care |
| Leadership Support | Statements indicating a provider's employer supports the use of telemedicine |
| Higher Quality of Delivered Care | Statements indicating better quality of care or regarding the growth of research on the efficacy of telemedicine |
| Ethics | Statements indicating that telemedicine is ethically appropriate or imperative |
| Supportive HIPAA Regulations | Statements indicating generally positive HIPAA regulations for telemedicine use |
| Supportive Laws | Statements indicating generally positive or supportive laws for telemedicine use |
| Supportive Prescription Regulations | Statements indicating the supportive nature of prescription regulations |
| Increased Safety (e.g., Limiting Contagion) | Statements indicating an increase in patient or provider safety as a direct result of telemedicine |
| Adequate Video/Audio Quality of Telemedicine Technology | Statements regarding that the technological requirements for telemedicine are currently adequate |
| Miscellaneous* | Statements indicating a facilitator but that were not codable in the other categories |

Note. The left-hand column denotes the categories into which the “Other [Please specify]” responses were coded. The right-hand column is a descriptor of each category. An * denotes a category that was added based on inductive qualitative analysis.
