# Supplementary material for: Barriers and facilitators to physicians’ telemedicine uptake during the beginning of the COVID-19 pandemic
Source: PLOS Digit Health. 2025 Apr 8;4(4):e0000818. doi: 10.1371/journal.pdig.0000818 (PMC11977993; doi:10.1371/journal.pdig.0000818)
Supplement: S2 Table — (DOCX) [file pdig.0000818.s002.docx]

**S2 Sample “Other” Responses**

| Participant Response | Response Given for Barrier or Facilitator | Coded Category |
| --- | --- | --- |
| Unclear at this point what the insurance reimbursement for these visits will be in the future/has been in this epidemic. | Barrier | Insufficient Insurance Reimbursement |
| Cannot do a physical exam | Barrier | Diminished Quality of Delivered Care |
| Will need improved broadband in rural areas | Barrier | Lack of Patient Access to Technology |
| Seeing patients who are too scared to leave their house | Facilitator | Better Patient Access to Care |
| Convenience for patience | Facilitator | Better Patient Access to Care |
| Decreased risk of disease transmission | Facilitator | Increased Safety |
| Nature of care being provided | Barrier | Was unable to code |
| Same comment as before | Facilitator | Was unable to code |
