# Supplementary material for: Barriers and facilitators to physicians’ telemedicine uptake during the beginning of the COVID-19 pandemic
Source: PLOS Digit Health. 2025 Apr 8;4(4):e0000818. doi: 10.1371/journal.pdig.0000818 (PMC11977993; doi:10.1371/journal.pdig.0000818)
Supplement: S3 Table — (DOCX) [file pdig.0000818.s003.docx]

**S3 Percentage Endorsed of Each Telemedicine Barrier and Facilitator**

| Barrier | Percentage |
| --- | --- |
| Frequently Endorsed (above 20%) | |
| Lack of Patient Access to Technology | 77.6 |
| Insufficient Insurance Reimbursement | 53.5 |
| Diminished Doctor-Patient Relationship | 46.9 |
| Inadequate Video/Audio Technology | 46.1 |
| Diminished Quality of Delivered Care | 42.1 |
| Potential for Medical Errors | 36.4 |
| Insufficient Telemedicine Training | 35.5 |
| Inefficient Use of Time | 20.6 |
| Moderately Endorsed (10-20%) | |
| Higher Risk to Patient Safety | 18.0 |
| Unsupportive HIPAA Regulations | 17.1 |
| Legal Concerns | 15.4 |
| Lack of Leadership Support | 14.0 |
| Unsupportive Prescription Regulations | 10.1 |
| Infrequently Endorsed (below 10%) | |
| Ethics | 8.8 |
| Higher Cost for Providers | 5.7 |
| Not Suitable for Certain Patients/Types of Care | 4.8 |
| Higher Cost for Patients | 2.2 |
| Inadequate Patient Technological Literacy | 1.8 |
|  | |
| Facilitator | Percentage |
| Frequently Endorsed (above 20%) | |
| Better Access to Care | 75.4 |
| Increased Safety | 70.6 |
| Efficient Use of Time | 60.5 |
| Lower Cost for Patients | 43.0 |
| Effectiveness | 28.9 |
| Adequate Video/Audio Technology | 28.1 |
| Lower Cost for Providers | 23.2 |
| Supportive HIPAA Regulations | 20.2 |
| Moderately Endorsed (10-20%) | |
| Leadership Support | 16.2 |
| Supportive Laws | 16.2 |
| Supportive Prescription Regulations | 14.5 |
| Better Doctor-Patient Relationship | 12.7 |
| Telemedicine Training I have Received | 10.1 |
| Ethics | 10.1 |
| Infrequently Endorsed (below 10%) | |
| Higher Quality of Delivered Care | 5.7 |
| Miscellaneous | 0.9 |
