# Supplementary material for: Barriers and facilitators to physicians’ telemedicine uptake during the beginning of the COVID-19 pandemic
Source: PLOS Digit Health. 2025 Apr 8;4(4):e0000818. doi: 10.1371/journal.pdig.0000818 (PMC11977993; doi:10.1371/journal.pdig.0000818)
Supplement: S4 Table — (DOCX) [file pdig.0000818.s004.docx]

**S4 Variables in Logistic Regressions for Barriers**

| Predictor | *b* | SE | Wald | *p* | Odds Ratio | Lower 95% | Upper 95% |
| --- | --- | --- | --- | --- | --- | --- | --- |
| Lack of Patient Access to Technology |  |  |  |  |  |  |  |
| Age | 0.019 | 0.017 | 1.247 | 0.264 | 1.019 | 0.986 | 1.054 |
| Man | -0.531 | 0.354 | 2.244 | 0.134 | 0.588 | 0.294 | 1.178 |
| White | -0.297 | 0.390 | 0.582 | 0.446 | 0.743 | 0.346 | 1.595 |
| **Medical Center** | **0.777** | **0.368** | **4.467** | **0.035** | **2.175** | **1.058** | **4.472** |
| Urban | -0.380 | 0.386 | 0.970 | 0.325 | 0.684 | 0.321 | 1.457 |
| Insufficient Insurance Reimbursement |  |  |  |  |  |  |  |
| Age | -0.015 | 0.014 | 1.255 | 0.263 | 0.985 | 0.959 | 1.012 |
| Man | -0.441 | 0.298 | 2.196 | 0.138 | 0.643 | 0.359 | 1.153 |
| White | 0.344 | 0.312 | 1.214 | 0.270 | 1.411 | 0.765 | 2.602 |
| Medical Center | -0.308 | 0.301 | 1.047 | 0.306 | 0.735 | 0.408 | 1.325 |
| Urban | 0.033 | 0.321 | 0.010 | 0.919 | 1.033 | 0.550 | 1.939 |
| Diminished Doctor-Patient Relationship |  |  |  |  |  |  |  |
| Age | 0.009 | 0.014 | 0.435 | 0.509 | 1.009 | 0.982 | 1.037 |
| Man | 0.485 | 0.299 | 2.629 | 0.105 | 1.624 | 0.904 | 2.92 |
| White | 0.584 | 0.319 | 3.353 | 0.067 | 1.793 | 0.960 | 3.349 |
| Medical Center | -0.246 | 0.303 | 0.657 | 0.417 | 0.782 | 0.432 | 1.417 |
| Urban | -0.013 | 0.321 | 0.002 | 0.967 | 0.987 | 0.526 | 1.852 |
| Inadequate Video/Audio Technology |  |  |  |  |  |  |  |
| Age | 0.008 | 0.014 | 0.379 | 0.538 | 1.009 | 0.982 | 1.036 |
| Man | -0.439 | 0.305 | 2.080 | 0.149 | 0.644 | 0.355 | 1.171 |
| White | 0.539 | 0.321 | 2.827 | 0.093 | 1.714 | 0.915 | 3.213 |
| **Medical Center** | **0.639** | **0.306** | **4.367** | **0.037** | **1.895** | **1.041** | **3.451** |
| Urban | -0.301 | 0.324 | 0.858 | 0.354 | 0.740 | 0.392 | 1.398 |
| Diminished Quality of Delivered Care |  |  |  |  |  |  |  |
| Age | 0.003 | 0.014 | 0.037 | 0.847 | 1.003 | 0.976 | 1.030 |
| Man | 0.002 | 0.299 | 0.000 | 0.996 | 1.002 | 0.557 | 1.800 |
| White | 0.055 | 0.312 | 0.031 | 0.860 | 1.057 | 0.573 | 1.947 |
| Medical Center | -0.184 | 0.301 | 0.374 | 0.541 | 0.832 | 0.462 | 1.500 |
| Urban | -0.079 | 0.318 | 0.062 | 0.804 | 0.924 | 0.495 | 1.724 |
| Potential for Medical Errors |  |  |  |  |  |  |  |
| **Age** | **-0.032** | **0.015** | **4.501** | **0.034** | **0.969** | **0.940** | **0.998** |
| Man | 0.181 | 0.312 | 0.336 | 0.562 | 1.198 | 0.650 | 2.210 |
| White | 0.100 | 0.327 | 0.093 | 0.761 | 1.105 | 0.582 | 2.095 |
| Medical Center | -0.386 | 0.314 | 1.519 | 0.218 | 0.679 | 0.367 | 1.256 |
| Urban | -0.231 | 0.327 | 0.498 | 0.480 | 0.794 | 0.418 | 1.507 |
| Insufficient Telemedicine Training |  |  |  |  |  |  |  |
| Age | -0.007 | 0.014 | 0.273 | 0.601 | 0.993 | 0.965 | 1.021 |
| Man | 0.355 | 0.307 | 1.342 | 0.247 | 1.427 | 0.782 | 2.602 |
| White | -0.212 | 0.320 | 0.440 | 0.507 | 0.809 | 0.432 | 1.515 |
| Medical Center | 0.312 | 0.311 | 1.011 | 0.315 | 1.367 | 0.743 | 2.513 |
| Urban | -0.238 | 0.333 | 0.512 | 0.474 | 0.788 | 0.411 | 1.513 |
| Inefficient Use of Time |  |  |  |  |  |  |  |
| **Age** | **-0.069** | **0.021** | **11.091** | **<.001** | **0.933** | **0.896** | **0.972** |
| **Man** | **1.011** | **0.374** | **7.326** | **0.007** | **2.749** | **1.322** | **5.718** |
| White | 0.347 | 0.425 | 0.667 | 0.414 | 1.415 | 0.615 | 3.254 |
| Medical Center | -0.182 | 0.374 | 0.237 | 0.627 | 0.834 | 0.401 | 1.734 |
| Urban | 0.472 | 0.432 | 1.195 | 0.274 | 1.603 | 0.688 | 3.736 |
