# Supplementary material for: Barriers and facilitators to physicians’ telemedicine uptake during the beginning of the COVID-19 pandemic
Source: PLOS Digit Health. 2025 Apr 8;4(4):e0000818. doi: 10.1371/journal.pdig.0000818 (PMC11977993; doi:10.1371/journal.pdig.0000818)
Supplement: S5 Table — (DOCX) [file pdig.0000818.s005.docx]

**S5 Variables in Logistic Regressions for Facilitators**

| Predictor | *b* | SE | Wald | *p* | Odds Ratio | Lower 95% | Upper 95% |
| --- | --- | --- | --- | --- | --- | --- | --- |
| Better Access to Care |  |  |  |  |  |  |  |
| Age | -0.009 | .015 | .313 | 0.576 | 0.991 | 0.962 | 1.022 |
| Man | -0.382 | 0.337 | 1.282 | 0.257 | 0.683 | 0.353 | 1.322 |
| White | 0.240 | 0.350 | 0.427 | 0.492 | 1.272 | 0.640 | 2.526 |
| Medical Center | 0.047 | 0.346 | 0.0196 | 0.891 | 1.048 | 0.532 | 2.065 |
| Urban | -0.003 | 0.370 | 0.000 | 0.993 | 0.997 | 0.482 | 2.059 |
| Increased Safety |  |  |  |  |  |  |  |
| Age | -0.009 | 0.015 | 0.405 | 0.525 | 0.991 | 0.962 | 1.020 |
| Man | -0.551 | 0.324 | 2.887 | 0.089 | 0.576 | 0.305 | 1.088 |
| White | 0.482 | 0.331 | 2.120 | 0.145 | 1.619 | 0.846 | 3.096 |
| Medical Center | 0.074 | 0.332 | 0.050 | 0.823 | 1.077 | 0.562 | 2.067 |
| Urban | 0.362 | 0.348 | 1.077 | 0.299 | 1.435 | 0.725 | 2.841 |
| Efficient Use of Time |  |  |  |  |  |  |  |
| Age | 0.019 | 0.014 | 1.753 | 0.186 | 1.019 | 0.991 | 1.048 |
| Man | -0.153 | 0.303 | 0.255 | 0.613 | 0.858 | 0.474 | 1.554 |
| White | -0.265 | 0.321 | 0.683 | 0.409 | 0.767 | 0.408 | 1.439 |
| Medical Center | 0.108 | 0.304 | 0.125 | 0.723 | 1.114 | 0.613 | 2.023 |
| Urban | 0.101 | 0.323 | 0.098 | 0.754 | 1.107 | 0.588 | 2.083 |
| Lower Cost for Patient |  |  |  |  |  |  |  |
| Age | -0.016 | 0.014 | 1.293 | 0.255 | 0.984 | 0.957 | 1.012 |
| Man | 0.562 | 0.307 | 3.355 | 0.067 | 1.754 | 0.961 | 3.199 |
| **White** | **-0.816** | **0.323** | **6.380** | **0.012** | **0.442** | **0.235** | **0.833** |
| **Medical Center** | **0.626** | **0.307** | **4.173** | **0.041** | **1.870** | **1.026** | **3.411** |
| Urban | 0.312 | 0.334 | 0.873 | 0.350 | 1.366 | 0.710 | 2.626 |
| Effectiveness |  |  |  |  |  |  |  |
| Age | 0.000 | 0.015 | 0.001 | 0.979 | 1.000 | 0.971 | 1.030 |
| Man | 0.046 | 0.328 | 0.020 | 0.889 | 1.047 | 0.551 | 1.991 |
| White | -0.378 | 0.331 | 1.311 | 0.252 | 0.685 | 0.358 | 1.309 |
| Medical Center | 0.095 | 0.332 | 0.082 | 0.775 | 1.099 | 0.574 | 2.106 |
| Urban | -0.440 | 0.345 | 1.630 | 0.202 | 0.644 | 0.328 | 1.265 |
| Adequate Video/Audio Technology |  |  |  |  |  |  |  |
| Age | 0.018 | 0.015 | 1.510 | 0.219 | 1.018 | 0.989 | 1.049 |
| Man | -0.316 | 0.335 | 0.890 | 0.346 | 0.729 | 0.379 | 1.405 |
| White | 0.201 | 0.350 | 0.330 | 0.566 | 1.222 | 0.616 | 2.425 |
| Medical Center | -0.037 | 0.332 | 0.012 | 0.911 | 0.964 | 0.503 | 1.846 |
| Urban | 0.159 | 0.354 | 0.202 | 0.653 | 1.172 | 0.586 | 2.347 |
| Lower Cost for Provider |  |  |  |  |  |  |  |
| Age | -0.024 | 0.017 | 1.919 | 0.166 | 0.977 | 0.945 | 1.010 |
| Man | 0.116 | 0.351 | 0.109 | 0.741 | 1.123 | 0.565 | 2.233 |
| White | -0.185 | 0.358 | 0.267 | 0.605 | 0.831 | 0.412 | 1.677 |
| Medical Center | -0.368 | 0.352 | 1.093 | 0.296 | 0.692 | 0.347 | 1.380 |
| Urban | 0.260 | 0.377 | 0.476 | 0.490 | 1.297 | 0.620 | 2.712 |
| Supportive HIPAA Regulations |  |  |  |  |  |  |  |
| Age | -0.012 | 0.017 | 0.478 | 0.489 | 0.988 | 0.956 | 1.022 |
| Man | -0.274 | 0.374 | 0.535 | 0.465 | 0.761 | 0.365 | 1.584 |
| White | 0.574 | 0.431 | 1.768 | 0.184 | 1.775 | 0.762 | 4.134 |
| Medical Center | 0.600 | 0.369 | 2.650 | 0.104 | 1.823 | 0.885 | 3.755 |
| Urban | 0.380 | 0.424 | 0.801 | 0.371 | 1.462 | 0.636 | 3.358 |
