# Supplementary material for: Barriers and facilitators to physicians’ telemedicine uptake during the beginning of the COVID-19 pandemic
Source: PLOS Digit Health. 2025 Apr 8;4(4):e0000818. doi: 10.1371/journal.pdig.0000818 (PMC11977993; doi:10.1371/journal.pdig.0000818)
Supplement: S6 Table — (DOCX) [file pdig.0000818.s006.docx]

**S6 Chi-squared and Nagelkerke pseudo-R squared**

| Category | Chi-squared | Degrees of Freedom | *p* | Nagelkerke pseudo-R squared |
| --- | --- | --- | --- | --- |
| Barriers | | | | |
| Lack of Patient Access to Technology | 8.013 | 5 | 0.156 | 0.053 |
| Insufficient Insurance Reimbursement | 7.179 | 5 | 0.208 | 0.041 |
| Diminished Quality of Doctor-Patient Relationship | 7.701 | 5 | 0.173 | 0.044 |
| Inadequate Audio/Video Technology | 9.429 | 5 | 0.093 | 0.054 |
| Diminished Quality of Delivered Care | 0.744 | 5 | 0.980 | 0.004 |
| Potential for Medical Errors | 7.311 | 5 | 0.199 | 0.043 |
| Insufficient Telemedicine Training | 2.890 | 5 | 0.717 | 0.017 |
| **Inefficient Use of Time** | **22.598** | **5** | **< 0.001** | **0.148** |
| Facilitators | | | | |
| Better Access to Care | 2.367 | 5 | 0.796 | 0.015 |
| Increased Safety | 6.374 | 5 | 0.272 | 0.039 |
| Efficient Use of Time | 2.843 | 5 | 0.724 | 0.017 |
| **Lower Cost for Patients** | **19.499** | **5** | **0.022** | **0.110** |
| Effectiveness | 2.845 | 5 | 0.724 | 0.018 |
| Adequate Audio/Video Technology | 2.213 | 5 | 0.819 | 0.014 |
| Lower Cost for Providers | 3.748 | 5 | 0.586 | 0.025 |
| Supportive HIPAA Regulations | 8.277 | 5 | 0.142 | 0.056 |
