# Supplementary material for: Barriers and facilitators to physicians’ telemedicine uptake during the beginning of the COVID-19 pandemic
Source: PLOS Digit Health. 2025 Apr 8;4(4):e0000818. doi: 10.1371/journal.pdig.0000818 (PMC11977993; doi:10.1371/journal.pdig.0000818)
Supplement: Attachment — Submitted filename: BF Physician Response Letter 09-24-24.docx [file pdig.0000818.s009.docx]

**Reviewer 1**

1. This original research manuscript entitled “Barriers and facilitators to physicians’ telemedicine uptake during the beginning of the COVID-19 pandemic” is a well written manuscript assessing providers attitudes towards telepsychology. Overall, the findings from the study are important and quite relevant in the current healthcare climate. I generally think this manuscript is a good one, though there are a few questions that arise when reading it. I generally think this manuscript is a good one, though there are a few questions that arise when reading it.

*Thank you for your comments and time spent reviewing.*

2. Who were the physicians enrolled in the study? It is not entirely clear. There is a section on Table 1 that would discusses the primary practice setting, but I am unclear what the specialties were. The reason this is important is that other data have suggested that specialty area of practice has influenced the uptake of TH.

*We have now added data on medical specialties in Table 2.*

3. The methods section is entirely too long and detracts from the findings of the manuscript. The investigative team should strongly consider revisions to this section. This is not a methods driven manuscript.

*We agree with this point and have shortened the Methods section by moving Table 2 (Final List of Barriers and Facilitators) in the original manuscript to the Appendix section (now S1) and removing the survey responses outlined in the text. We also have now dramatically cut down on the Procedure subsection of the Method, making it three sentences.*

4. The results section is a bit hard to get through with the embedded tables. But these tables are the actual important findings that should have a bit more text added to the results section.

*Based on similar feedback from Reviewer 2, we have moved Tables 2-6 to the Appendix section and bolded the statistically significant relationships to improve readability and understanding. We have now added and labelled p-values in the text and added the following language in the first paragraph of the Results section: “The sample included 228 physicians who were an average of 46.14 years old (SD=10.12). A greater proportion of the physicians were women (64%), identified as White (75%), and were practicing in urban and suburban settings (92.5%). There was low racial/ethnic diversity among physicians and a low percentage of physicians working in rural areas. The sample characteristics are summarized in Table 1.”*

5. Given the uncertainty of the participant specialties, the conclusions may be a bit overly broad.

*We have now updated our Conclusion section to tie more closely into the implications of the study findings.*

**Reviewer 2**

1. This article surveyed a range of physicians in the US to determine their views on the use of telemedicine at the beginning of the COVID Pandemic. This is an important and relevant question as the role of telemedical consults has grown recently. Their questions were based on literature review and are appropriate. The sampling process to recruit subjects for the survey had limitations, although using a broader survey on mental health care to de-emphasize the telemedicine aspect could have strengthened the recruitment of a broader range of practitioners not just those interested in technology. Overall this paper includes valuable insights but there are a number of issues that need to be addressed.

*Thank you for your comments. We appreciate your time reviewing our paper.*

2. Abstract: Good, on first reading. Clear relevant points, not structured. More information is needed on sampling methods, and lack of racial and ethnic representation. Authors should include the statistical analyses used and the significant results found. More information is needed on sampling methods, and lack of racial and ethnic representation. Authors should include the statistical analyses used and the significant results found.

*We have now updated our Abstract by adding information on sampling methods, demographics of the sample, statistical analyses, and significant results.*

3. Introduction: Good clear statement of research questions. Survey was sent to 850 by email, 46 were undelivered, 315 opened survey, 228 with full data were included. There was a low percentage of black and Latino physicians and those from rural areas. Details of survey participants should be in the results section. Survey occurred at the beginning of the COVID pandemic so likely reflected experiences before COVID and initial scale up with existing technologies.

*We have now described our sample characteristics in the Results section and pointed out the lack of racial/ethnic representation in the sample. We have added the following language in the first paragraph of the Results section: “The sample included 228 physicians who were an average of 46.14 years old (SD=10.12). A greater proportion of the physicians were women (64%), identified as White (75%), and were practicing in urban and suburban settings (92.5%). There was low racial/ethnic diversity among physicians and a low percentage of physicians working in rural areas. The sample characteristics are summarized in Table 1.”*

4. Results: Table 1: Needs N in table. Percentages only need 1 decimal place. Percentage calculations are wrong, for example in section on Race/ethnicity and section on Primary Practice Setting. Full table needs to be recalculated and redesigned for clarity.

*We have now redesigned the table for clarity and recalculated the percentages and moved Table 1 to the Results section.*

5. Table 2: Structure needs to be improved to link titles and responses clearly (table lines?) The table is large and gives no indication of the frequency of different coded responses. It would be clearer to summarize the most common types of free text responses in the results and include a full table of all responses and numbers in each category in an appendix.

*We have moved Table 2 (now S1) to the Appendix section. The percentage of endorsed codes can be found in S2.*

6. Tables 4 and 5: These tables are largely redundant as few of the odds ratios are significant at the P< .05 level. They should be summarized in the text or in much smaller tables that just highlight the significant values and perhaps indicates those that are of borderline significance. The full tables could be in appendices. It would be helpful to indicate to the reader that Exp(B) is the odds ratio.

*We have moved Table 4 (now S3) and Table 5 (now S4) to the Appendix section and bolded the statistically significant relationships to improve readability and understanding. We have also labelled p-values in the text and changed Exp(B) to “Odds Ratio” for clarity.*

7. Table 6: This table only shows 2 statistically significant relationships and could be simplified with clear highlighting of the significant relationships, or moved to an appendix with significant results in the text.

*We have moved Table 6 (now S3) to the Appendix section and bolded the two statistically significant relationships.*

8. Discussion: Clear and helpful with important points about limitations to the survey. The sampling process to recruit participants clearly had limitations in terms of responses. Do the authors have any evidence as to whether this may have favored certain groups?

*This is an excellent point. We have now added the following language to the Limitations section: “Though current study’s sampling process had a response rate comparable to many similar studies, certain groups of physicians may have been more likely to complete the study, such as those with more flexible time to do so, those whose email addresses were a part of common medical listservs, and those who were scientifically minded and open to participating in research. Future studies should include a larger more diverse sample with multiple sampling approaches (e.g., in person, physical mail, advertisements, etc.) to aid in generalizability.”*

9. Do the authors believe the results would be different 1-2 years after the start of the pandemic when the technology, training, experience and legal and economic structures had changed?

*We have expanded our literature review for the Discussion section and added findings from studies that were conducted at the beginning of the pandemic as well as 1-2 years after the start of the pandemic.*

10. Limitations of the statistical analyses also need to be discussed particularly the relatively small sample size for the logistic regression analysis and the small number of statistically significant relationships.

*We have now added the following language: “The statistical analyses may also have been limited because of the relatively small sample size for the logistic regression analyses and possibly as a result the small number of statistically significant relationships. A larger sample of physicians may have been able to uncover additional statistically significant relationships, although they likely would have been small-sized and possibly not as robust or important as those found in the current study.”*

**Reviewer 3**

1. Thank you for submitting your manuscript on barriers and facilitators to physicians' telemedicine uptake during the COVID-19 pandemic. Your study provides valuable insights into this very intense topic. The mixed-methods approach and substantial sample size are strengths. However, there are several areas that require attention before publication.

*Thank you for taking the time to review this manuscript and for offering your comments.*

2. The structure could be improved by moving the explanation of survey options earlier in the methods section.

*We have moved Table 2 (now S1) to the Appendix section based on Reviewer 2’s feedback. The first reference to the explanation of survey options is now appearing earlier in the Methods section (i.e. the last sentence of the Measures sub-section).*

3. The results presentation, particularly Table 4, is overwhelming and could benefit from a more digestible format, such as smaller tables or visual representations.

*We have moved Table 4 (now S3) and Table 5 (now S4) to the Appendix section, bolded the statistically significant relationships, and reformatted the tables to improve readability and understanding.*

4. While the discussion is comprehensive, it could be strengthened by more explicit connections to prior literature and deeper exploration of policy implications.

*We agree with this point. In the first and second paragraphs of the Discussion section, we have now expanded our literature review by connecting our findings to prior literature on barriers and facilitators of telemedicine and added policy implications associated with sustained telemedicine adoption.*

5. The limitations section is candid but could further address how these limitations might influence result interpretation, especially regarding the COVID-19 context's impact on generalizability.

*We have now added the following language to the first paragraph of the Limitations section: “Since data were collected at the beginning of the COVID-19 pandemic, it is important to contextualize the findings and recognize the transformative role telemedicine played during the critical transition period for the healthcare field. As a result, generalizability of the findings is limited, as certain perceived barriers or facilitators identified by physicians at the beginning of the pandemic may be less relevant in the post-COVID era (e.g., virtual visits facilitated infection prevention and control).”*

6. Additionally, the low Nagelkerke pseudo-R squared values warrant more explicit discussion. Addressing these points will enhance the manuscript's clarity, rigor, and impact. We look forward to seeing a revised version that builds on the strong foundation you've established.

*We have now added the following in the Discussion section: “The Nagelkerke pseudo-R squared statistics in the logistic regression models had a wide range, suggesting that the predictors explained 0.4%-14.8% of the variance in barrier endorsement and between 1.4%-11.0% of the variance in facilitator endorsement. As a result, these model effect sizes were largely in the small- (≤0.01) to medium- (≤0.09) sized range and suggested the variables holistically were predictive of barrier or facilitator endorsement.”*
