# Supplementary material for: Barriers and facilitators to physicians’ telemedicine uptake during the beginning of the COVID-19 pandemic
Source: PLOS Digit Health. 2025 Apr 8;4(4):e0000818. doi: 10.1371/journal.pdig.0000818 (PMC11977993; doi:10.1371/journal.pdig.0000818)
Supplement: Attachment — Submitted filename: BF Physician Response Letter 02-20-25.docx [file pdig.0000818.s010.docx]

**Reviewer 4**

1. Please add a statement about data availability. As mentioned in this journal, PLOS Data policy requires authors to make all data underlying the findings described in their manuscript fully available without restriction, with rare exception. The data should be provided as part of the manuscript or its supporting information, or deposited to a public repository.

*We have now added the Data Availability Statement at the end of the Method section: “**The study’s anonymized data are available in the online supplementary files for the article.” Although in our previous submission we made the data publicly available in the supporting files, it does not appear that the editorial management system allowed the reviewer access to it.*

**Reviewer 5**

1. This is an interesting manuscript that explores potential clinician-perceived barriers and facilitators or telehealth at the start of the pandemic. Although somewhat dated, it is still valuable to know what demographics may be particularly likely or unlikely to adopt telehealth to inform education efforts.

*Thank you for your comments and the time spent reviewing.*

2. Previous reviewer comments on the unclear structure of the results and the inclusion of excessive detail have been satisfactorily addressed. However, some concerns remain to do with the relevance of findings in the 2025 context (and how well the discussion addresses this) and limitations of the qualitative approach. The paragraph beginning with “Identifying the most and least endorsed barriers and facilitators can help inform institutional policies” requires more nuanced consideration given much has changed since the time of the survey. This should be a larger focus of the discussion, not just relegated to the limitations section. I’m aware that you also commented about the consistency of the findings during the pandemic, but post-pandemic there have been many changes in reimbursement, access to improved technology, improving digital literacy, literature on the lack of impact of digital delivery on quality of care or therapeutic alliance. Your discussion could be strengthened by consideration of which factors are likely to persist in the current environment as that will be more informative for the design of interventions.

*We have now drastically expanded our Discussion section to increase the relevance of our findings in the 2025 context. We have reframed our discussion by incorporating post-pandemic changes in in reimbursement, access to improved technology, improving digital literacy, literature on the lack of impact of digital delivery on quality of care or therapeutic alliance to allow for more nuanced interpretations of the findings. The limitations of the qualitative approach have also now been addressed in Reviewer 5 Comment #7 below.*

3. Qualitative methodologies are broad and you have not specified what approach was used (thematic? Content?) or the coding approach taken (semantic? Inductive? Deductive?)?

*In the second paragraph of the Data Analysis sub-section, we have now specified that we used content analysis as our qualitative methodology: “To appropriately code the qualitative “other” responses for both advantages/facilitators and barriers/deterrents, we conducted a content analysis of the participants’ responses [21] that is similar to the descriptive content methods used in prior qualitative research on barriers and facilitators to telepsychology use. [22]”*

*In the same paragraph, we provided detailed clarification on the coding approach taken: “We used a blended deductive and inductive coding procedure. [23,24] The original 16 barrier categories and 15 facilitator categories were generated by researchers with extensive expertise in the field of telehealth technologies and relied on prior literature on the most frequently researched barriers and facilitators to telemedicine. This research framework was used to identify these categories, establish guidelines for the types of responses that would fall under each category, and appropriately code participant responses into the researcher generated categories. Further, an inductive coding approach was used to derive concepts and themes that extended beyond the original 31 researcher generated categories. This resulted in the addition of 2 barrier/deterrent categories and 1 advantage/facilitator category.”*

4. Which staff were raters? Without adequate description of the qualitative methods it is hard to judge quality and alignment with existing approaches.

*In the second paragraph of the Data Analysis sub-section, we have now specified that the initial raters and the third rater/tiebreaker were all psychology PhD students.*

5. It seems potentially problematic to simply recode the free text responses under the existing options - presumably, the respondents felt like these didn’t adequately capture their perspectives, hence they provided an “other” response. And this lacks some of the richness that would be expected of a qualitative report. I found the absence of example comments disappointing as they could provide useful elaboration, for example, on why the quality of care or the relationship was impacted.

*We have now acknowledged our approach of recoding the free text responses under the existing options as a limitation in the Limitations sub-section in Reviewer 5 Comment #7. We have created a table entitled “S6 Sample ‘Other’ Responses” in Supporting Information files, which contains example free text comments provided by the physicians.*

6. I’m not sure why the “miscellaneous” category was included, what does this add to the analysis when there is no coherent idea linking these responses?

*We created the miscellaneous category for codes that did not appear to fit into the main themes, as suggested by Braun & Clarke (2006). This methodology has also been used in a study on telepsychology use (Watson et al., 2023). Thus, we used this residual category to house codes that were deemed not common enough to generate a new thematic category or did not fit into the deductive categories in our coding framework. We have now clarified the reason why we included the “miscellaneous” category at the end of the second paragraph of the Data Analysis sub-section: “The miscellaneous category was created for codes that did not fit into the main themes. [25] This residual category was used to house codes that were either deemed not common enough to generate a new thematic category or did not fit into the deductive categories in our coding framework. This method of categorizing codes has been used in previous studies on telehealth use. [22]”*

7. A potential limitation is integrating the free text responses into the quantitative analysis. If a physicians didn’t mention, for example, inadequate patient technological literacy in a free text comment, it doesn’t mean they do not perceive this to be important. It may have simply meant they were not motivated enough or too time poor to comment. They may have selected these if they were presented in the survey. This may give the reader an inaccurate impression that this barrier was not perceived as important - rather, it was not asked after. This should be acknowledged as a limitation especially in light of literature showing the impacts of the digital divide on access to healthcare.

*We agree with the reviewer and have now added this as a limitation to the* *Limitations and Future Directions sub-section: “Another limitation of the study is that participants’ free text responses were integrated into the existing themes, which impacted the richness and nuances of the qualitative findings. For example, while inadequate technological literacy was not listed in the survey as a barrier for physicians to choose from, it does not mean that this barrier was not perceived as important by the physicians; if it were presented in the survey, some physicians might have selected it. Future research may consider conducting qualitative interviews with physicians to examine their perceptions and opinions of telemedicine use with a focus on the impacts of the digital divide on access to telemedicine.”*

**Reviewer 6**

1. Great work on addressing the comments and improving the readability of the paper!

*Thank you for your positive comment and time spent reviewing.*

2. Minor thoughts/questions: * Participants (p. 7): What was the geographical focus of this study or what physicians in the USA did you invite to participate? - might be worth mentioning this first thing to not cause any confusion later on (since the N would be quite small for all US physicians).

*We recruited physicians of all specialties across the U.S. irrespective of geographical location. We have now clarified this point in the beginning of the first paragraph of the Participants sub-section: “**Physicians of all specialties were recruited across the U.S., irrespective of specific geographic location within the U.S.”*

3. Procedures (p. 7): Since that's a short sub-section and somewhat overlaps with previous information, I wondered if it would be useful to merge this section with "participants"?

*We have now merged the Procedure sub-section with the Participants sub-section.*

4. Data Analysis (p. 8): What does "endorsing" barriers/facilitators mean? Merely selecting them in the survey (and those items that were not selected were automatically "not endorsed")?

*Yes, the reviewer is correct. We took selecting a barrier/facilitator in the survey to mean endorsement of that barrier/facilitator. We have now clarified this point in the first paragraph of the Data Analysis sub-section: “Endorsement of barriers and facilitators was defined as survey respondents choosing each option with select-all-that-apply. The options not selected were automatically considered as not endorsed by the respondents.”*

5. Results (p. 12): are the statistical measures you present a p-value for ORs? (e.g.: "Older physicians were less likely to endorse inefficient use of time (p < .001) and potential for medical errors (p = 0.034) as barriers to telemedicine use compared to younger physicians." - it might be worth mentioning the measurement as they are part of the supplementary material but not the "main paper".

*Yes, these are p-values. A previous reviewer from the first round of reviews asked us to move all of our tables—and therefore main quantitative findings—to supplementary documents. To balance their request with us providing some level of statistical descriptors, we included p-values in text whenever describing statistical effects. We have now specified in the first p-value appearance that they are “for the individual odds ratio.”*

6. Discussion (p. 18): Personal request: Would you mind mentioning "allied health professionals" instead of singling out PTs in the last sentence of the discussion?

*We have now replaced “physical therapists” with “allied health professionals.”*

**Reviewer 7**

1. Thank you for the opportunity to review this manuscript describing barriers and facilitators for the use of telemedicine by physicians at the beginning of the COVID-19 pandemic. The authors have tackled an important topic, provided a strong rationale for the investigation, and thoughtful discussion of the implication of results. They have improved this manuscript through revisions and suggestions from reviewers and it appears easier to read and under\stand findings. Although I do believe this manuscript overall would add to the body of literature for this topic, I believe that there are several areas that could be revised to enhance clarity and ensure the manuscript accurately reflects the findings, given the statistical analyses used. Thus, my comments are largely focused on the results section and ensuring that significant findings are appropriately explained in the context of all analyses conducted. I believe these revisions will strengthen the manuscript and allow readers to better interpret these findings. Below, I have provided detailed feedback organized by section for the authors.

*Thank you for your careful reading of our manuscript, your kind words regarding our work, and your thoughtful feedback. We respond to each of your specific points below.*

2. The introduction may benefit from slight restructure to include the importance of telemedicine and factors associated with sustained use (lines 97 through 103) prior to review of barriers and facilitators of use (lines 88 through 96). This would help improve and establish the rationale for examining these factors and their importance (even as we move into “post” COVID-19 healthcare).

*These lines have now been moved as the reviewer suggested.*

3. Methods- Do you have more information regarding the sampling procedures used? I wonder what percentage of the 850 recruitment announcements sent were individuals identified through hospital and health clinic websites and were these hospitals national or local to the researcher’s institution? How were these hospitals and health clinics selected? This may help explain and understand the physician characteristics and help understand generalizability to other physicians in the United States.

*In response to reviewer 5, Question 2 we have added the following sentence to the open of the Participants section: “Physicians of all specialties were recruited across the U.S., irrespective of specific geographic location within the U.S.” We do not have a record of which physicians were recruited via what information depository; however, we do specify in the next sentence of the Participants section that all physicians were recruited “using directories of hospital and health clinic websites, professional organizations, social media groups, and professional newsgroups.”*

4. Results - What does it mean if the omnibus binary logistic regressions were not significant? Does that impact interpretation of the predictors?

*We agree with the reviewer’s point here. There is debate in the literature about what it means if individual predictors in a regression are significant but the omnibus test for the overall regression is not. Typically, it means that the sample was just underpowered to detect the omnibus effect, which we believe to be the case in the current study. As a result, we have now noted in the Limitations section that sample size may have impacted the power of the study, thereby limiting our ability to detect some smaller individual or omnibus effect sizes. We also now state in the Discussion section the following: “The Nagelkerke pseudo-R2 statistics in the logistic regression models had a wide range, suggesting that the predictors explained 0.4%-14.8% of the variance in barrier endorsement and between 1.4%-11.0% of the variance in facilitator endorsement. As a result, these model effect sizes were largely in the small- (<0.01) to medium- (<0.09) sized range. Only two of the overall models including all relative study predictors (age, gender, race, medical practice location, and urbanicity) were significant. The relatively low explanatory power of the models may be due to (a) a lack of critical predictors in the model, (b) low statistical power due to the smaller sample size, or (c) the inclusion of predictors that do not add significant explanatory power to the model. Thus, despite the relative lack of significance for the majority of the models, we examined the strength of individuals predictors.”*

5. Given these are multivariable logistic regressions, statements of results and significance of variables (e.g., age) should be stated “within the context of all other variables included in the model”.

*You are correct. Thank you! We have now added this phrasing to the paragraph discussing individual odds ratios.*

6. There were 16 separate logistic regressions run in this study. 4 out 8 barrier analyses yielded no significant results and 7 of 8 facilitator analyses yielded no significant results. Please add explanation to the results section detailing null findings and comment in the discussion as to why these predictors may not have explained variation in endorsement of a particular barrier/facilitator.

*Our preference in empirical journal articles is typically not to detail large numbers of null findings (other than explicitly showing they are null as we do in our tables) unless there are specific null findings that run contrary to previous literature or theory. There are many reasons for null findings: (a) there may not actually be an effect to pick up on; (b) the effect might be too small given the study’s power; (c) the items or scales that tap the constructs may have inherent measurement error due to imprecise wording; or (d) there may be additional unknown methodological confounds obscuring real results. Unfortunately, researchers from null results alone rarely know which of these options is the case. As a result, we believe that in the current study with a good number of null findings, a thorough discussion of them would be more methodologically obscuring than illuminating. We have now directly addressed this point in the Limitations section of the manuscript: “**This study returned a number of non-significant predictors of barriers and facilitators to telemedicine uptake. There could be manifold reasons for the lack of significant findings including a true lack of effect, poor statistical power, item or scale constructs may have inherent measurement error due to imprecise wording, or there may be additional unknown methodological confounds obscuring real results. Future research may wish to investigate these predictors in a larger sample with greater power.”*

7. Discussion- Be careful with language on line 236. It seems like identifying barriers and facilitators could inform institutional policies aimed at increasing adoption or sustainment of use of telemedicine… but I’m not sure about funding.

*We have removed this statement.*

8. The interpretation of the Nagelkerke pseudo-R2 statistics (lines 251 through 253) is misleading, as only two models were significant (and meaningfulness is up for interpretation or defense). This should be revised to be more accurate.

*We have deleted a reference to the models holistically predicting barriers and facilitators to help attenuate the language. We have also changed the following lines to be more descriptive in nature and to more accurately reflect our intended interpretation of the results: “**The Nagelkerke pseudo-R squared statistics in the logistic regression models had a wide range, suggesting that the predictors explained 0.4%-14.8% of the variance in barrier endorsement and between 1.4%-11.0% of the variance in facilitator endorsement. As a result, these model effect sizes were largely in the small- (≤0.01) to medium- (≤0.09) sized range, with two overall models having significant predictive capability which included the demographic predictors of age, gender, race, medical practice location, and urbanicity.”*

9. Table S1 is still confusing, particularly the note. The table displays all codes used in the study, but the note makes it appear that these were all generated based on qualitative responses. Consider providing a general description of table and then specific notes including more information for clarity (e.g., “Descriptions of barriers and facilitators created based on research team agreement. Physicians’ “other” responses resulted in 20 facilitators and 43 barriers that were coded into the following categories: lack of patient access (n = 9), …”).

*We have adjusted the table considerably following recommendations across this and the two subsequent comments. We have also added some details regarding the table, categories, and coding methods throughout the Methods section. We hope these changes assist in making the table clearer.*

10. Denote on Table S1 the three categories added to the list of researcher-generated codes.

*We have now added a notation indicating which of the categories were added during the qualitative portion of the analysis.*

11. Consider landscape format for Table S1, consistency in text alignment within cells, and adding spacing to increase readability.

*We have reformatted Table S1 to be justified (for even spacing) and added all borders to improve readability. Please also note that the journal will adjust this based on their house style beyond our control.*

**Reviewer 8**

1. This paper provides useful insight into the barriers and facilitators, and contextual characteristics influencing these factors, associated with telemedicine use in the US. The authors have addressed reviewer comments well however, some revisions to reviewer comments could be addressed better and there are a couple of minor additional points that should be considered.

*Thank you for the time you spent reading our manuscript and for your kind words and thoughtful feedback.*

2. Addressing previous reviewer comments: while the methods section has been slightly shortened, the description of the qualitative analysis could be further shortened/clarified to explain what the raters did i.e. what qualitative analysis strategy did the authors use? Looking at the reference it seems to be a qualitative content analysis. This could be presented much more simply e.g. the raters conducted independent content analysis to identify and categorise additional barriers/facilitators. Also the results of this qualitative analysis should be presented in the results rather than the methods.

*In response to several reviewers’ comments, we have now incorporated greater detail on the qualitative analysis methodology while streamlining the methods section to the best of our ability given the requests for additional information by multiple reviewers.*

3. The authors have moved the main results tables to supplementary materials per previous reviewers comments that the tables were too long, however now there are no main results tables presented in the results section. It would be useful to have a shorter table to substantiate claims on some of the main/significant findings (e.g. S2), and then the supplementary materials could be referenced for full results.

*In response to the general feedback from previous reviewers, we have removed the results tables from the main document and now have added additional references to statistical results throughout the Results section and discussion. These can be seen in the last two paragraphs of the results section and the second paragraph of the Discussion section. Given some of the conflicting feedback from reviewers, we hope this compromise is amenable.*

4. Additional points relating to methods (data analysis section): a) the grouping of practice characteristics requires further explanation/justification. For medical centers vs other settings, please clarify what the definition of medical centers was in the current study e.g. was a hospital considered a medical center? For urban vs suburban and rural settings, please either justify why suburban and rural were grouped or perhaps consider referencing the grouping of these settings as a limitation. Typically, urban and suburban would be grouped as rural settings possess unique characteristics. Also, the results could highlight that there were no stat significant results for urban vs suburban and rural (and limitations could highlight that this could be due to the urban/suburban not being much different contextually).

*We have now added the following details to the methods section to clarify predictor groupings: “For these groupings, medical center was defined as either an academic medical center or a Department of Veterans Affairs medical center, and suburban physicians were grouped with rural physicians were grouped primarily due to the sample size in an attempt to detect unique features related to urbanicity (as opposed to rural and suburban characteristics).”*

*We initially re-wrote the results section to include a highlight of the lack of statistical significance for urban vs suburban/rural, but we feel this made the results section imbalanced given that no other statistically non-significant results were discussed. We have however listed the choice to group suburban and rural together in the Limitations and Future Directions section as it fits much more parsimoniously there: “The lack of response from rural providers made the grouping of practice location (urban vs suburban and rural) difficult. While the current study did not uncover a significant predictive relation between urbanicity and the various barriers and facilitators, it is possible that, had the rural sample been larger, we might have uncovered significant results for rurality (rural vs urban/suburban).”*

5. Additionally, the data analysis section does not describe why chi squared tests and Nagelkerke pseudo-R squared were run, some brief additional detail on this would be useful.

*We have expanded our descriptions of the logistic regression in the data analysis section to include the reasons for the chi-squared and Nagelkerke pseudo-R squared test: “Chi-squared and Nagelkerke pseudo-R squared statistics were calculated to test the overall significance of the logistic regression models with all predictor variables.”*

**Reviewer 9**

1. This study examines telemedicine adoption during the COVID-19 pandemic, a critical period of healthcare transformation. The analysis of key barriers and facilitators offers practical insights for healthcare policy and telemedicine implementation strategies. However, the manuscript requires significant revisions before it can be considered for publication:

*Thank you for your recognition of the significance and novelty of our work!*

2. Logistic Regression Models- The paper does not adequately explain why specific variables were chosen for the logistic regression models, weakening the rationale behind the analysis.

*In response to several of the other reviewers’ comments, we have significantly more detail regarding how categories and predictors were selected based off their use in previously literature in both telemedicine and telepsychology research.*

3. The logistic regression models have low explanatory power (e.g., low Nagelkerke pseudo-R² values), indicating that critical factors influencing barriers and facilitators might be missing.

*We have now included a direct reference to the overall models in the results section. Further, based on the feedback here and from Reviewer 7, Question 4, we have greatly expanded the discussion regarding the overall models: “**The Nagelkerke pseudo-R squared statistics in the logistic regression models had a wide range, suggesting that the predictors explained 0.4%-14.8% of the variance in barrier endorsement and between 1.4%-11.0% of the variance in facilitator endorsement. As a result, these model effect sizes were largely in the small- (≤0.01) to medium- (≤0.09) sized range. Only two of the overall models including all relative study predictors (age, gender, race, medical practice location, and urbanicity) were significant. The relatively low explanatory power of the models may be due to (a) a lack of critical predictors in the model, (b) low statistical power due to the smaller sample size, or (c) the inclusion of predictors that do not add significant explanatory power to the model. Thus, despite the relative lack of significance for the majority of the models, we examined the strength of individuals predictors.*”

4. Important variables such as years in practice, the number of providers in a practice, and additional practice settings were not included, despite their potential relevance to telemedicine adoption. The authors should incorporate these variables and explicitly justify their inclusion or exclusion.

*We thought about including each of these predictors, but they either were fully multicollinear with other predictors (e.g., years in practice being multicollinear with age, number of providers in a practice with practice setting such as hospital) or did not have enough individual subgroups to meaningfully include as a predictor (e.g., additional practice settings).*

5. Discussion Section- The findings are not sufficiently grounded in prior literature, reducing the strength of the discussion.

*Both the Introduction and Discussion sections have now been greatly extended to offer a deeper analysis of existing literature and to tie the results, where appropriate, to recent research on telehealth, telemedicine, and telepsychology.*

6. Specific Gaps in Citations: Lines 221-227: No references are provided to support differences in telemedicine adoption between older and younger physicians.

*There is currently no research available on why age might impact difference in telemedicine uptake following the COVID-19 pandemic. As such, we have added the following phrase to our discussion of age-related differences: “however, no research to date has explored why this discrepancy might exist.”*

7. Lines 232-236: The claim that physicians may be frustrated by the lack of in-person resources or that telemedicine reduces patient costs due to avoided ancillary fees lacks supporting citations.

*At present, no research to date has investigated this possibility. Therefore, we have added the following statement to this section of the discussion: “however, no research to date has investigated this possibility.” Further, we have added the following section to the Limitations and Future Directions section in hopes of highlighting the need for additional investigation: “Finally, the research on barriers and facilitators to telehealth is still in its relative infancy, especially after the COVID-19 transformation of telehealth services. This study highlighted a number of gaps in the literature regarding what might underpin provider suspicion of or affinity for telemedicine. Future research may wish to examine, for instance, why male providers perceive of telemedicine as an inefficient use of time or whether physicians believe telemedicine appointments routinely lack vital resources that might be available for in-person visits.”*

8. Lines 240-248: While the authors appropriately cite literature on female providers spending more time with patients, they do not provide evidence for gender-based differences in telemedicine appointment durations or implementation challenges.

*This section has been restructured to highlight the limited research on gender-based differences in telemedicine preference (that female providers spend more time with patients), while also offering other possible but untested explanations. “Male physicians were more likely to endorse inefficient use of time as a barrier than female physicians. There is some research suggesting female providers also take longer on average with their patients than male providers, [39] suggesting more willingness to have a longer patient encounter. The additional time needed to start a telemedicine appointment may be especially salient for male providers. Additional research on gender-based differences in telemedicine preference is severely lacking leaving us to hypothesize other possible explanations. Male physicians might generally prefer in-person visits or grow impatient more quickly with troubleshooting difficulties, resulting in a perception that telemedicine wastes time. Patient distractibility (e.g., providing care for a child while in a telemedicine appointment) may also be influencing the difference between male and female providers. It is possible that the way in which the telemedicine program is implemented may add additional burden to the process (e.g., a telemedicine clinic that enables providers from a major metropolitan medical center to treat patients who are still required to go in-person to a rural telemedicine medical center as opposed to a direct telemedicine to home program).”*

*As noted in an earlier response to comments, we have also added the following section to the Limitations and Future Directions section to highlight the need for investigation of the mechanism underpinning provider preference (or lack thereof) for telemedicine: “Finally, the research on barriers and facilitators to telehealth is still in its relative infancy, especially after the COVID-19 transformation of telehealth services. This study highlighted a number of gaps in the literature regarding what might underpin provider suspicion of or affinity for telemedicine. Future research may wish to examine, for instance, why male providers perceive of telemedicine as an inefficient use of time or whether physicians believe telemedicine appointments routinely lack vital resources that might be available for in-person visits.”*

9. Minor Feedback- Remove categories/levels with N=0, %=0 in Table 1 (e.g., Correctional Facility, Geriatric Facility).

*We have now deleted all categories/levels with N=0%, %=0 in Table 1.*

10. Separate Tables 4 and 5 into individual tables with titles reflecting outcomes (e.g., "Better Access to Care"). Move chi-squared and Nagelkerke pseudo-R² values from Table 6 into corresponding tables for logistic regression results.

*Thank you for this feedback. We attempted to do as you requested, creating 17 individual tables, one for each logistic regression reported in the results section. Once we did, however, we felt that the tables became unwieldy as a result, especially when referencing so many tables within the body of the manuscript. Given the conflicting feedback we have received from reviewers (one wanted fewer/shorter tables while one wanted an additional table added, some wanted tables in the text while another wanted them all moved to supplemental materials) we have attempted to incorporate as much of the feedback as possible from all reviewers. In the hopes of meeting feedback from multiple reviewers, we have modified Table 1 significantly, added specific references to the table as well as excerpts of the statistics within the body of the manuscript, and added an entirely new table (Table 2). We hope that this compromise is amenable as we try to juggle the somewhat conflicting feedback.*

11. By addressing these issues, the manuscript will better justify its methodology, improve the integration of findings into existing literature, and enhance its clarity and impact.

*Thank you for your feedback and suggestions. We agree that the manuscript is in a much better condition as a result of incorporation your feedback and that of the other reviewers.*
